# Dendritic cells in tumor microenvironment promoted the neuropathic pain via paracrine inflammatory and growth factors

**DOI:** 10.1080/21655979.2020.1771068

**Published:** 2020-06-17

**Authors:** Zhun Wang, Kai Song, Wenxin Zhao, Zhongmin Zhao

**Affiliations:** a Department of Pain Management, Tianjin First Center Hospital, Tianjin, China; b Department of Anesthesiology, Tianjin Medical University NanKai Hospital, Tianjin, China; c School of the Fourth Clinical Medicine, Capital Medical University, Beijing, China; d Department of Pain Management, Hospital Affiliated 5 to Nantong University (Taizhou People’s Hospital), Taizhou, China

**Keywords:** Cancer, neuropathic pain, dendritic cells, inflammatory factors, growth factors, dorsal root ganglia, Wnt signaling, PDGF, TNF, NRG1

## Abstract

Neuropathic pain associated with cancers was caused by tumor itself or tumor therapy, which was aggravated by sensitizing nociceptor sensory neurons. The tumor microenvironment contributed to tumorigenesis, tumor progress, tumor metastasis, tumor immune resistance, tumor chemotherapy, and tumor immunotherapy. In the current study, we explored the contributions of the infiltrated dendritic cells insulted by Wnt1 in tumor microenvironment to neuropathic pain associated with cancers. The different transcriptome of infiltrated dendritic cells from lung adenocarcinoma and from juxtatumor indicated that thousands of genes were up-regulated by the tumor microenvironment, some of which were enriched in pain pathway. The paracrine factors such as TNF, WNT10A, PDGFA, and NRG1 were also elevated in tumor-infiltrating dendritic cells. The receptors of paracrine factors were highly expressed on dorsal root ganglia (DRG), and not altered in pain conditions. Single-cell RNA-seq data unveiled that TNFSF1 was expressed in neurons, microglial cells, and endothelial cells. PDGFRA was only expressed in microglial cells. ERBB3 was only expressed in neurons. FZD1 and 3 were extensively expressed in various cells. The components composed of signaling pathways associated with the above paracrine factors participated in pain networks. The transcription factors activated by paracrine factor signaling regulated the expression of genes associated with pain. TNF, WNT10A, and PDGFA were extensively expressed in multiple cancers, but their expression in patients did not distribute normally. These data indicated that infiltrated dendritic cells in tumor microenvironment promoted neuropathic pain by sensitizing nociceptor sensory neurons via paracrine factors. Blockage of paracrine factor signaling might alleviate cancer pain.

## Graphical abstract

**Figure:**
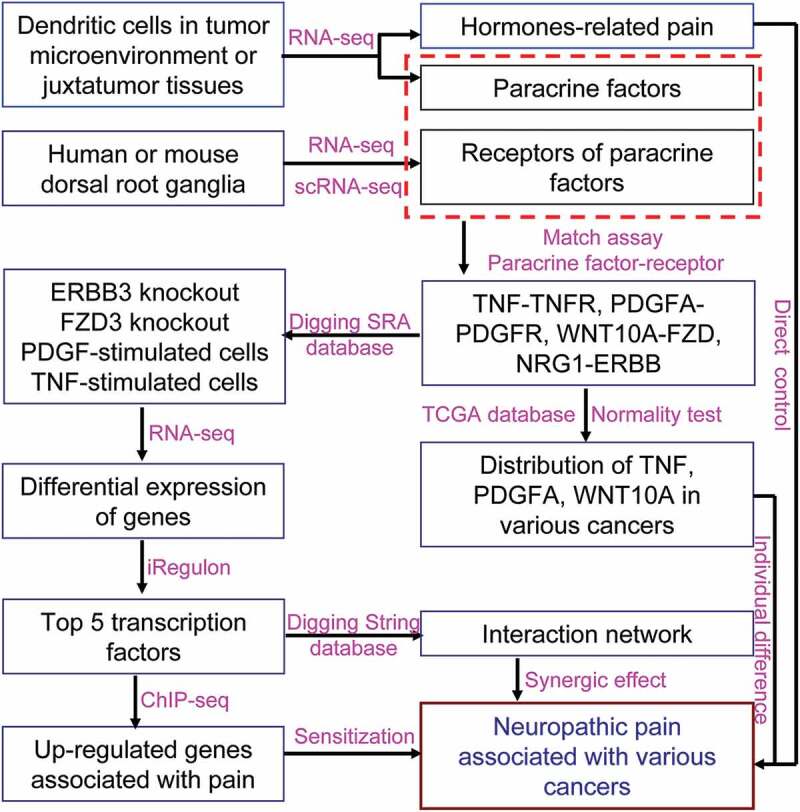

## Introduction

Cancer pain is one of the most common complications in cancer patients. More than 70% of the cancer patients suffered from pain [1–3]. Cancer pain is dependent on the type of cancers, the stage of the disease, and the pain threshold of the person. Cancer pain can result from cancer itself or from the treatment of cancer. For instance, tumor growth pressed the nerve and resulted in cancer pain. Chemotherapy and radiotherapy damaged the nerve which caused neuropathic pain in cancer patients. Neuropathic pain was aggravated by the sensitized nociceptor sensory neurons. Cytokines and growth factors such as interleukin 1 beta (Il-1β) and nerve growth factor (NGF) had been reported to sensitize nerves [4], but the detailed mechanisms are still not fully understood. For example, the proteins involved in neuropathic pain which were regulated by sensitizing signaling were not clear. The transcription factors activated by sensitizing signaling which regulated the expression of genes associated with neuropathic pain needed to be identified. Except for Il-1β and NGF, other novel factors and their mediated-pathways which sensitized nerves needed to be unveiled.

Recently single-cell RNA-seq (scRNA-seq) discovered that tumor microenvironment composed of dozens of cell populations, where cell-cell commutation regulated the cell behavior and function [5,6]. ScRNA-seq unveiled that a module of CD8(+) tumor-infiltrating lymphocytes were distinct dysfunctions related to intracellular metallothioneins and a zinc-finger transcription factor gata-3 due to their interaction with tumors [6]. Extensive research indicated that tumor microenvironment not only contributed to tumorigenesis, tumor progress, tumor metastasis, and tumor immune resistance but also was associated with the antitumor effect of chemotherapy and immunotherapy [7–13]. The co-operation of several types of cells including macrophages, neutrophils, T and B cells, fibroblasts, pericytes, and adipocytes which are located in the tumor microenvironment that promoted tumor angiogenesis through various contributions such as secretion of growth factors, degradation of perivascular extracellular matrix [12]. Simultaneously these cells locating in the tumor microenvironment regulated tumor growth, progress, and metastasis by directly interacting with tumor cells or indirectly secreting soluble factors [7]. The developed medicines that targeted tumor microenvironment to enhance cancer treatment include ramucirumab, an inhibitor of vascular endothelial growth factor receptor in endothelial cells, and emactuzumab, an antibody targeting colony-stimulating factor-1 receptor in macrophages [9]. However, as we know, there were not many reports about the effect of various cells such as dendritic cells located at tumor microenvironment on cancer pain.

In the current study, we tried to explore the contributions of the tumor microenvironment to cancer pain, and identify the potential mechanisms including novel factors, factor-mediated pathways, pathway-related transcription factors (TF), and TF-regulated genes associated with pain. In order to achieve the goal, the dendritic cells which infiltrated into lung adenocarcinoma were chosen as an investigating example because they were dysfunctional after interaction with Wnt1 (WNT stands for wingless-related integration site) derived from tumor cells [14]. Using RNA-seq data from dendritic cells infiltrated into tumors or the juxtatumor tissues [14], the genes in dendritic cells infiltrated into tumors that were associated with synthesis or metabolism of pain-related hormones were up-regulated significantly, indicating that the dendritic cells might directly regulate cancer pain via hormones synthesis. More importantly, the paracrine factors derived from infiltrated dendritic cells such as tumor necrosis factor (TNF), platelet-derived growth factor-alpha (PDGFA), WNT10A, and neuregulin 1 (NRG1) were increased significantly, while scRNA-seq data from dorsal root ganglia (DRG) indicated that the increased paracrine factors might sensitize nociceptor sensory neurons in DRG by interacting with their receptors to aggravate neuropathic pain associated with cancers. The downstream transcription factors such as transcription factor 7 Like 1 (TCF7L1), chromodomain helicase DNA binding protein 1 (CHD1), and B-Cell lymphoma 3-encoded protein (BCL3) activated by signaling from the interaction between paracrine factors and their receptors might promote expression of genes associated with pain including arrestin beta 2 (ARRB2), cyclin-dependent kinase 5 (CDK5), and cannabinoid receptor 1 (CNR1). These novel findings unveiled the new factors and new mechanisms that tumor microenvironment would participate in regulating cancer pain.

## Methods and material

### Enrichment assay

Enrichment assay was performed using the PANTHER tools (http://pantherdb.org/) [15,16]. Briefly, the genes were inputted and the organism was selected. Analysis method was designed to use the statistical overrepresentation test. Finally, the analysis category such as PANTHER pathway was determined. The reference gene list was selected according to the organism. Fisher’s exact test was used to judge the statistical difference. False discovery rate (FDR) was used to correct the statistical results. P < 0.05 was significant different in statistical analysis. The enrichment index was calculated as the ratio of actual genes to expected genes.

### DRG RNA-seq data extraction

The human dorsal root ganglia (DRG) samples from organ donors and the orthologous mouse DRG samples were used to perform RNA-seq analysis. The data were deposited in the website (https://bbs.utdallas.edu/painneurosciencelab/sensoryomics/drgtxome/) [17]. The detailed data were extracted out by keyword searching. To compare the gene expression of pain condition to normal control, the entire RNA-seq data of human DRGs from six individual donors were downloaded [17]. Expression of receptors associated with cytokines, chemokines, and growth factors, which were identified to be potentially involved in cancer pain mediated by infiltrated dendritic cells, was extracted and used to analyze the effect of pain on their expressions.

### RNA-seq data analysis

The RNA-seq data for dendritic cells were SRX5170813, SRX5170814, SRX5170815, SRX5170816, SRX5170817, SRX5170818, SRX5170819, SRX5170820, SRX5170821, SRX5170822, SRX5170823, SRX5170824, SRX5170825, and SRX5170826. The RNA-seq data for erythroblastic leukemia viral oncogene homologue 3 (ERBB3) knockout and wild type control samples were SRX5281766, SRX5281767, SRX5281770, and SRX5281771. The RNA-seq data from PDGF stimulation and vehicle control cells were SRX4650630, SRX4650633, SRX4650636, SRX4650629, SRX4650631, and SRX4650635. The RNA-seq data for Frizzled Class Receptor 3 (FZD3) knockout and wild type control samples were SRX1539877, SRX1539878, SRX1539879, SRX1539883, SRX1539884, and SRX1539885. The RNA-seq data for TNF stimuli and vehicle control cells were SRX6092588, SRX6092589, SRX6092584, and SRX6092585. All original RNA-seq data were downloaded from SRA database. The original reads were trimmed using FASTX-toolkit (V 0.0.13, http://hannonlab.cshl.edu/fastx_toolkit/index.html) and aligned to reference genome using TopHat2 (V2.1.1, https://ccb.jhu.edu/software/tophat/index.shtml). The differential expression analysis was performed using Cuffdiff (V2.2.1, http://cole-trapnell-lab.github.io/cufflinks/cuffdiff/). The different data from dendritic cells were used to evaluate the alteration of the transcriptome of tumor-infiltrated dendritic cells compared with juxtatumor dendritic cells. The different data from other cells or tissues were used to identify the top five transcription factors activated by paracrine factors and their target genes using iRegulon.

### Network establishment

All networks were established using Cytoscape software [18]. The network for pain was generated by keyword pain in String disease query with confident cutoff of 0.5 and the protein number of 1000 [19]. The network was used for enrichment assay in Cytoscape (3.7.2, https://cytoscape.org/). The sub-networks were generated from the full pain network by using TNF, WNT10A, and NRG1 as keywords in Cytoscape. The network for top five transcription factors was generated using all identified transcription factors as keywords in String protein query with a confident cutoff of 0.5 and the protein number of 100.

### Single-cell RNA-seq assay

The single-cell RNA-seq data matrix from mouse DRG was downloaded from GEO (GSE71453, https://www.ncbi.nlm.nih.gov/geo/download/?acc=GSE71453&format=file&file=GSE71453%5FDRG%5FSNT%5F3%2D7days%5FTPM%5Fgeo%2Ecsv%2Egz). The data matrix was read into R (V3.6.1, https://www.r-project.org/), and then analyzed in Seurat package (Searat 3.0, https://satijalab.org/seurat/) [20]. Briefly, the cells with low and high RNA counts, and high mitochondria genes were excluded. The matrix was normalized, and then used to identify highly variable features. The normalized matrix was scaled, and then used to perform linear dimensional reduction with principal component analysis (PCA). The final matrix was used to cluster cells, and perform non-linear dimensional reduction with uniform manifold approximation and projection (UMAP). Finally, the different markers among clusters were found to define the cell populations. Violin plots were used to display the expression of receptors of cytokines, chemokines, and growth factors.

### Identification of transcription factors

Transcription regulators including transcription factors were predicted by iRegulon in Cytoscape [21]. The network was established in Cytoscape using differential expression genes from knockout or stimulated samples. The promoter regions (10 kb around, transcription start site, TSS) of genes in the network were screened by motifs (9713 position weight matrix, PWMs) and tracks (1120 ChIP-seq tracks) of transcription factors. The recovery standard conditions were an enrichment score threshold more than 3.0 and receiver operating characteristic curve (ROC) threshold less than 0.03. The transcription factors with top five net expression score (NES) were used to further investigate their effects on gene expression associated with pain.

### ChIP-seq data and displaying

ChIP-seq data were downloaded from GEO with format bw, bigwig, or bed. The ChIP-seq data for transcription Factor 7 Like 1 (TCF7L1), SAM pointed domain-containing ETS transcription factor (SPDEF), chromodomain helicase DNA binding protein 1 (CHD1), RE1 silencing transcription factor (REST), E2F transcription Factor 1 (E2F1), suppressor of zeste 12 protein homolog (SUZ12), B-Cell lymphoma 3-encoded protein (BCL3), histone H3 acetylation at lysine 27 (H3K27Ac), and histone H3 trimethylation at lysine 4 (H3K4Me3) were GSE80337, GSM1187121, GSM1573654, GSM1782714, GSM2501567, GSM3937782, GSM1010775, GSM3937775, and GSM3937778, respectively. The ChIP-seq data were loaded into Integrative Genomics Viewer (IGV, https://igv.org) [22,23]. Hg19 was chosen as the reference genomics. The genes associated with pain were input into searching to locate them in genomics, and their regulation by transcription factors was displayed and viewed. After properly narrowed the gene region, the windows were snapped to make pictures.

### Statistical analysis

Statistical differences between multiple groups were determined by one- or two-way ANOVA with Bonferroni post hoc multiple analysis. Two-group comparisons were analyzed by the two-tailed-unpaired Student’s *t* test. P < 0.05 was statistically significant. The normal distribution was evaluated using D’Agostino and Pearson omnibus normality test. All statistical analysis was performed using GraphPad Prism (V. 8.0)

## Results

### Transcriptome alteration of dendritic cells in tumor microenvironment

**Figure 1.**
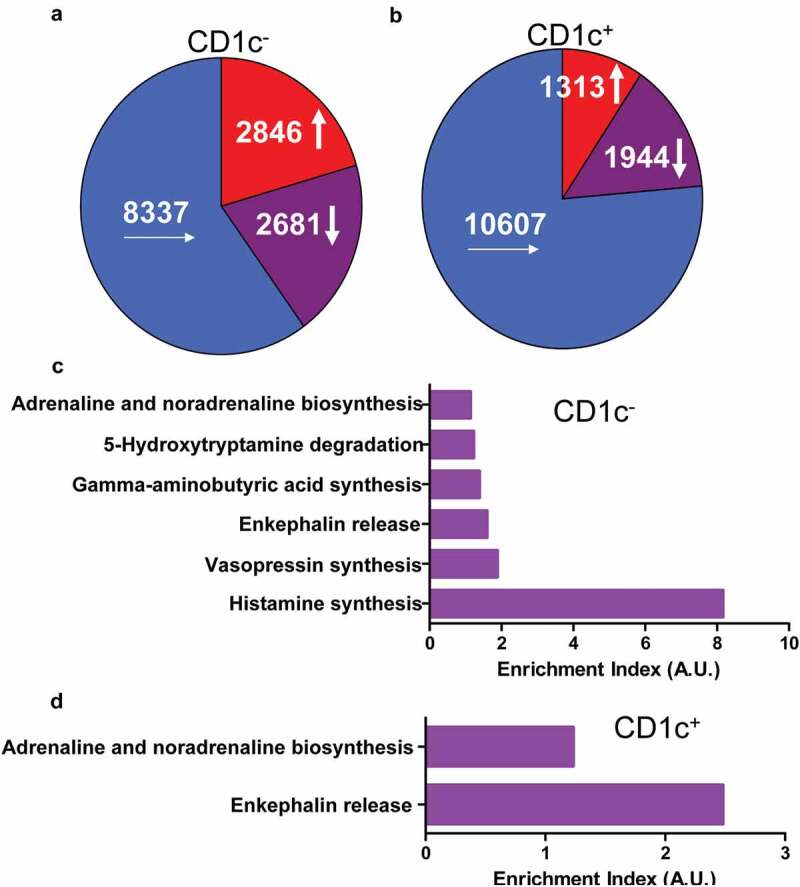
Genes were up-regulated in tumor-infiltrated dendritic cells and enriched in pathways associated with pain. The dendritic cells were isolated from tumor or juxtatumor tissues, and RNA was extracted from dendritic cells and used to RNA-seq. (a) The transcriptome of tumor-infiltrated CD1c^−^ dendritic cells. (b) The transcriptome of tumor-infiltrated CD1c^+^ dendritic cells. (c) The enrichment of genes from 1.5-fold up-regulated genes of tumor-infiltrated CD1c^−^ dendritic cells in pain associated pathways. (d) The enrichment of genes from 1.5-fold up-regulated genes of tumor-infiltrated CD1c^+^ dendritic cells in pain associated pathways. Enrichment index was calculated as ratio of actually up-regulated genes to expectedly up-regulated genes. A.U. was arbitrary unit.

Recently single-cell RNA-seq unveiled that several dozens of cell populations located at tumor microenvironments which were associated with tumor immune escape, immunotherapy, and chemotherapy [7–13]. There was no report about the relationship between cell populations and neuropathic pain related to cancers yet. Comparison of transcriptomes of CD1c negative (CD1c^−^) and positive (CD1c^+^) dendritic cells which infiltrated into lung adenocarcinoma with those located at juxtatumor indicated that there were 2846 (Figure 1(a)) and 1313 (Figure 1(b)) genes that had more than 1.5-fold increase in expression, respectively. Pathway analysis indicated that the highly expressed genes were enriched in pathways associated with pain such as adrenaline and noradrenaline biosynthesis, 5-hydroxytryptamine degradation, gamma-aminobutyric acid synthesis, enkephalin release, vasopressin synthesis, and histamine synthesis (Figure 1(c,d)). These results suggested that dendritic cells insulted by paracrine factors or cancers might synthesize and release the peptides and hormones associated with pain.

**Figure 2.**
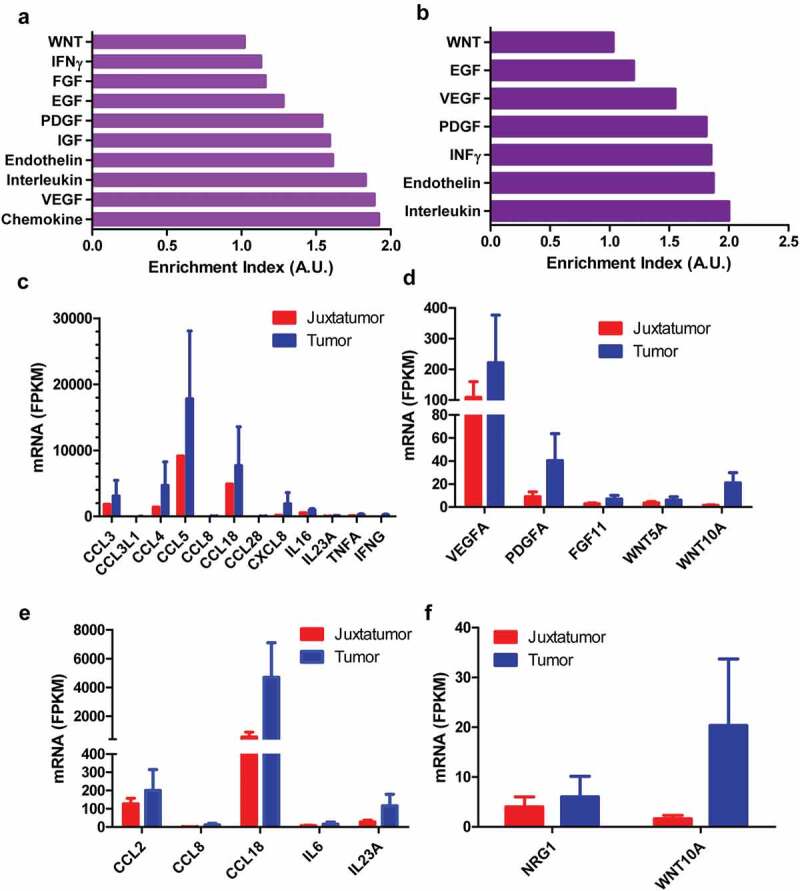
The higher expression paracrine factors from tumor-infiltrated dendritic cells. The dendritic cells were isolated from tumor or juxtatumor tissues, and RNA was extracted from dendritic cells and used to RNA-seq. (a) and (b). The enrichment of cytokines, chemokines, growth factors, and other paracrine factors from tumor-infiltrated CD1c^−^ (a) and CD1c^+^ dendritic cells. Enrichment index was calculated as the ratio of actually up-regulated genes to expectedly up-regulated genes. A.U. was arbitrary unit (c) and (d). The increased expression of cytokines and chemokines (c) as well as growth factors and other paracrine factors (d) in tumor-infiltrated CD1c^−^ dendritic cells compared with juxtatumor CD1c^−^ dendritic cells. (e, f) The increased expression of cytokines and chemokines (e) as well as growth factors and other paracrine factors (f) in tumor-infiltrated CD1c^+^ dendritic cells compared with juxtatumor CD1c^+^ dendritic cells.

Besides enrichment of highly expressed genes in pathways directly associated with pain, these genes were also enriched in pathways associated with cytokines and chemokines as well as growth factors (Figure 2(a,b)). Comparison with dendritic cells from juxtatomor tissues, chemokines such as CCL2, CCL3, CCL3L1, CCL4, CCL5, CCL8, CXCL8, cytokines such as IL6, IL16, IL23A, TNFA, INFG, growth factors such as VEGFA, PDGFA, FGF11, NRG1, and other paracrine factors such as WNT5A and WNT10A were higher in dendritic cells that infiltrated into tumors (Figure 2(c–f)). For example, the average FPKM of PDGFA and WNT10A was 40.3 and 21.0, respectively, in infiltrated dendritic cells while the average FPKM was only 9.0 and 1.5 in juxtatumor dendritic cells, respectively (Figure 2(c)). These liquid factors might interact with their receptors in neurons to regulate pain.

### Receptors in DRG responded to paracrine factors from dendritic cells

**Figure 3.**
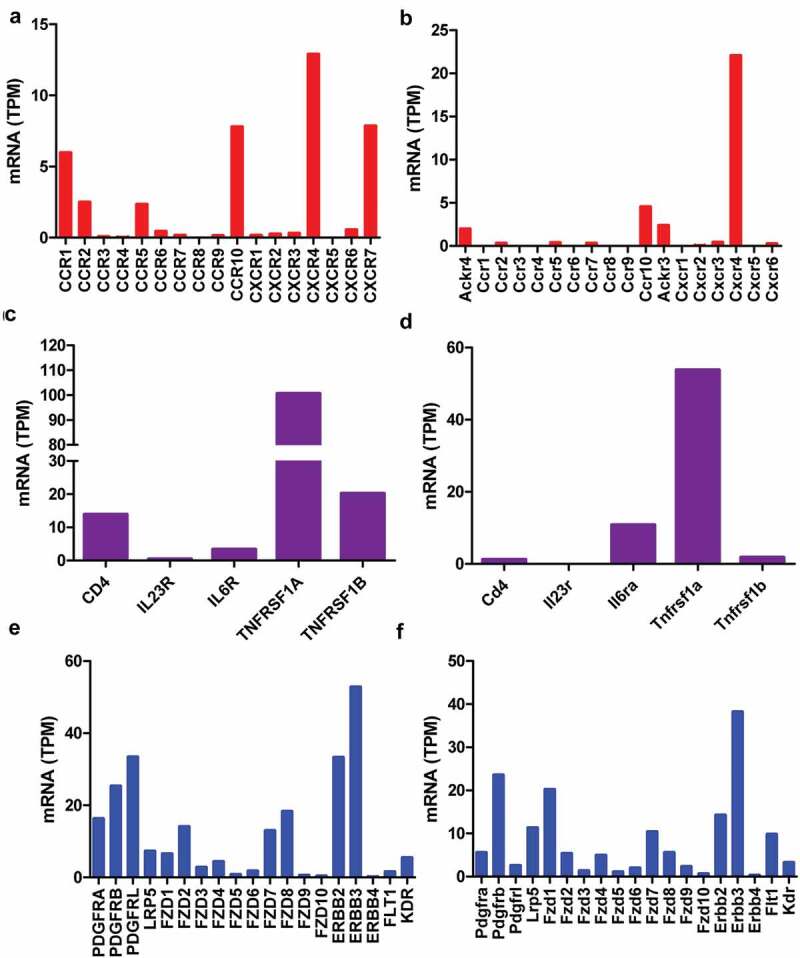
Receptors of paracrine factors in human and mouse dorsal root ganglia (DRG). RNA-seq data indicated that chemokine receptors (a, b), cytokine receptors (c, d), growth factors and other paracrine factors (e, f) in human (a, c, e) and mouse (b, d, f) DRG. The data showed mean of three samples.

**Figure 4.**
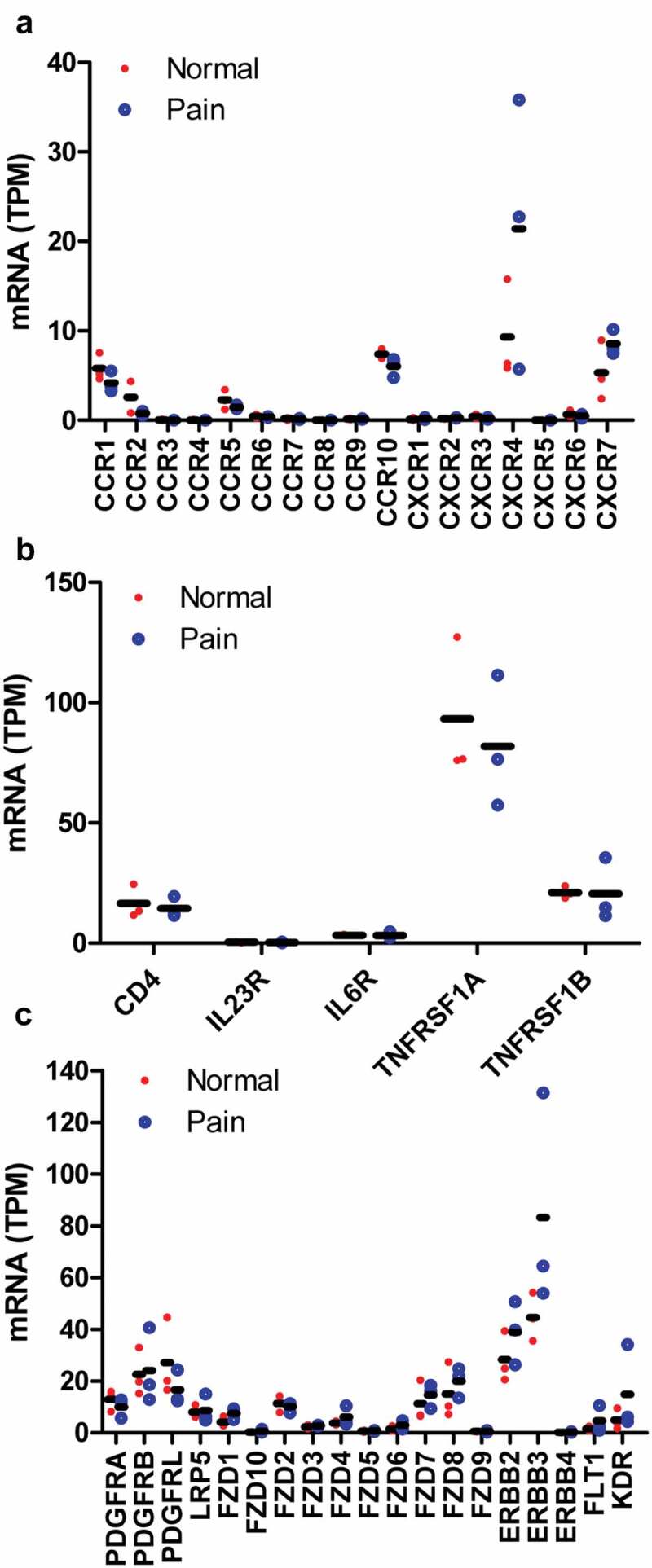
The expression alteration of paracrine factor receptors under pain conditions. RNA-seq data indicated that chemokine receptors (a), cytokine receptors (b), growth factor receptors and other paracrine factor receptors (c) were not altered in pain patients compared with normal control. The data showed the individual value of three samples and their mean.

The dorsal root ganglia (DRG) contain proprioceptive, low-threshold, and damage-sensing nociceptive sensory neurons. Thus, the receptors of cytokines, chemokines, growth factors, and other paracrine factors were determined using RNA-seq data from human and mouse DRG. Among the chemokine receptors only CCR10 (7.8 and 4.5 TPM) and CXCR4 (12.9 and 22.0 TPM) have high expression in both human and mouse DRG, respectively (Figure 3(a,b)), and are not altered under pain condition (Figure 4(a)). The other chemokine receptors were less than CCR10 and CXCR4 within the same species (Figure 3(a,b)), especially in mice, other chemokine receptors were lower than 3 TPM (Figure 3(b)). However, CCR10 ligand CCL27 and CCL28 as well as CXCR4 ligand CXCL12 were not detectable in infiltrated dendritic cells (data not shown). These data excluded the possibility that interaction between chemokines and their receptors was involved in pain mediated by infiltrated dendritic cells. Among the cytokine receptors, TNFRSF1A (100.6 and 53.8 TPM), a TNF receptor, has high expression in both human and mouse DRG, respectively (Figure 3(c,d)). TNFRSF1A did not change under pain condition (Figure 4(b)). TNFA in infiltrated dendritic cells (280.5 FPKM) was 2.5-fold in juxtatumor dendritic cells (115.8 FPKM) (Figure 2(a)). These data suggested that TNF–TNFRSF1A interaction was involved in pain associated with dendritic cells.

Further examination of growth factor receptors, PDGF receptor A (16.3 and 5.6 TPM) and B (25.3 and 23.6 TPM) as well as NRG1 receptor ERBB2 (33.3 and 14.3 TPM) and ERBB3 (52.8 and 38.2 TPM), has high expression in both human and mouse DRG, respectively (Figure 3(e,f)). They did not alter under pain condition (Figure 4(c)). WNT receptor FZD1 (6.5 and 20.2 TPM), FZD2 (14.1 and 5.3 TPM), FZD7 (12.3 and 10.4 TPM) and FZD8 (18.3 and 5.9 TPM) have higher expression than other members including FZD3 (2.8 and 1.4 TPM), FZD4 (4.6, and 4.7 TPM), FZD5 (0.8 and 1.1 TPM), FZD6 (1.8 and 2.0 TPM), FZD9 (0.6 and 2.3 TPM), and FZD10 (0.4 and 0.7 TPM) in both human and mouse DRG, respectively (Figure 3(e,f)). They did not change under pain condition (Figure 4(c)). PDGFA, NRG1, and WNT10A in infiltrated dendritic cells were 4.5-, 1.5-, and 14-fold in juxtatumor dendritic cells (Figure 2(d,f)). These data suggested that PDGF-PDGFR, NRG1-ERBB, and WNT-FZD interaction might be involved in pain associated with dendritic cells.

**Figure 5.**
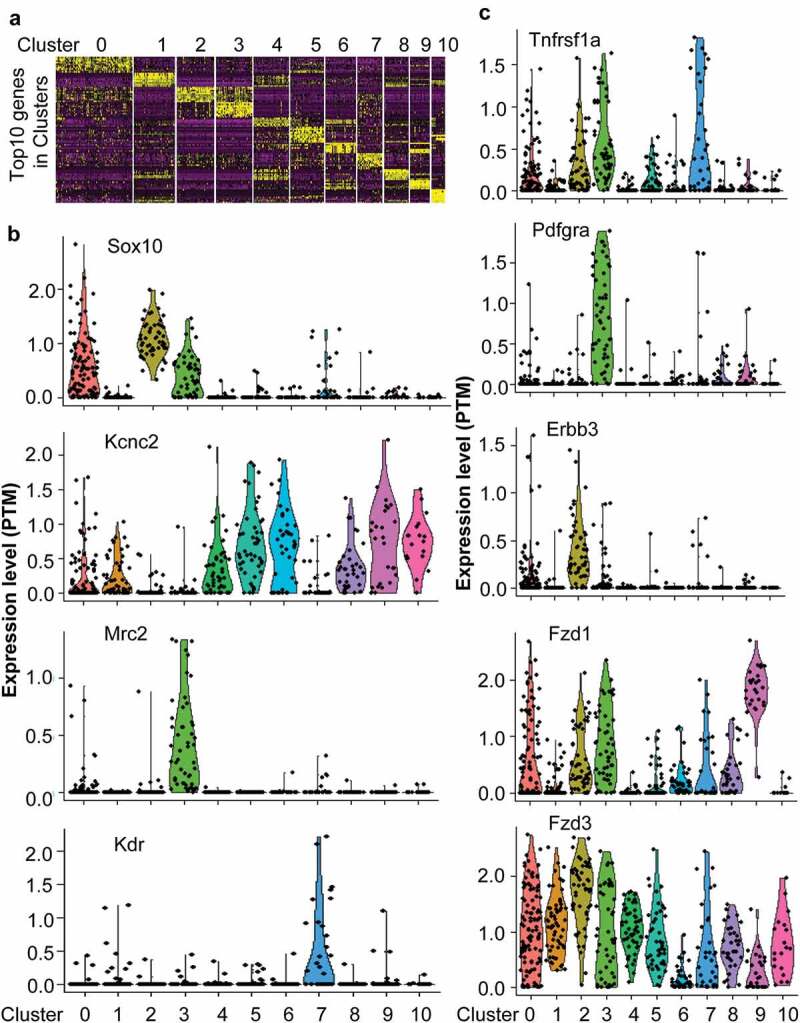
The single-cell RNA-seq data unveiled receptor expression in different subpopulation cells. (a) Single-cell RNA-data indicated that DRG composed of 11 clusters labeled from 0 to 10. (b) The specific markers to define different subpopulation cells. (c) The expression of paracrine factor receptors including Tnfrsf1a, Pdgfra, Erbb3, Fzd1, and Fzd3 in different subpopulation cells.

In order to determine which subpopulation of cells would be targeted by paracrine factors from infiltrated dendritic cells, single-cell RNA-seq data from DRG were used to define subpopulation cells and receptor distribution. The single-cell RNA-seq data indicated that DRG was composed of 11 subpopulation cells which labeled from cluster0 to cluster10 (Figure 5(a)). Cluster0 and cluster2 were Sox10 positive neurons (Figure 5(b)). Cluster3 was Sox10 and Mrc2 positive microglial cells (Figure 5(b)). Cluster1, 4–6, and 8–10 were Kcnc2 positive neurons (Figure 5(b)). Cluster7 was Kdr positive endothelial cells (Figure 5(b)). Tnfrsf1a was expressed on Sox10 positive cluaster0 and 3 neurons, Kcnc2 positive cluster6 neuron, Mrc2 positive cluster3 microglial cells, and Kdr positive endothelial cells (Figure 5(c)). Pdgfra was expressed on Mrc2 positive cluster3 microglial cells (Figure 5(c)). Krbb3 was expressed on Sox10 positive cluster0 and 2 neurons (Figure 5(c)). FZD1 and FZD3 were extensively expressed on almost all cell subpopulations (Figure 5(c)). These data indicated that paracrine factors such as TNF, PDGFA, and WNT10A from infiltrated dendritic cells would directly target neurons or microglial cells via their receptors to regulate pain.

### The interaction between paracrine factors such as TNF, WNT and NRG1, and their receptors including TNFR, ERBB, and FZD participated in pain

**Figure 6.**
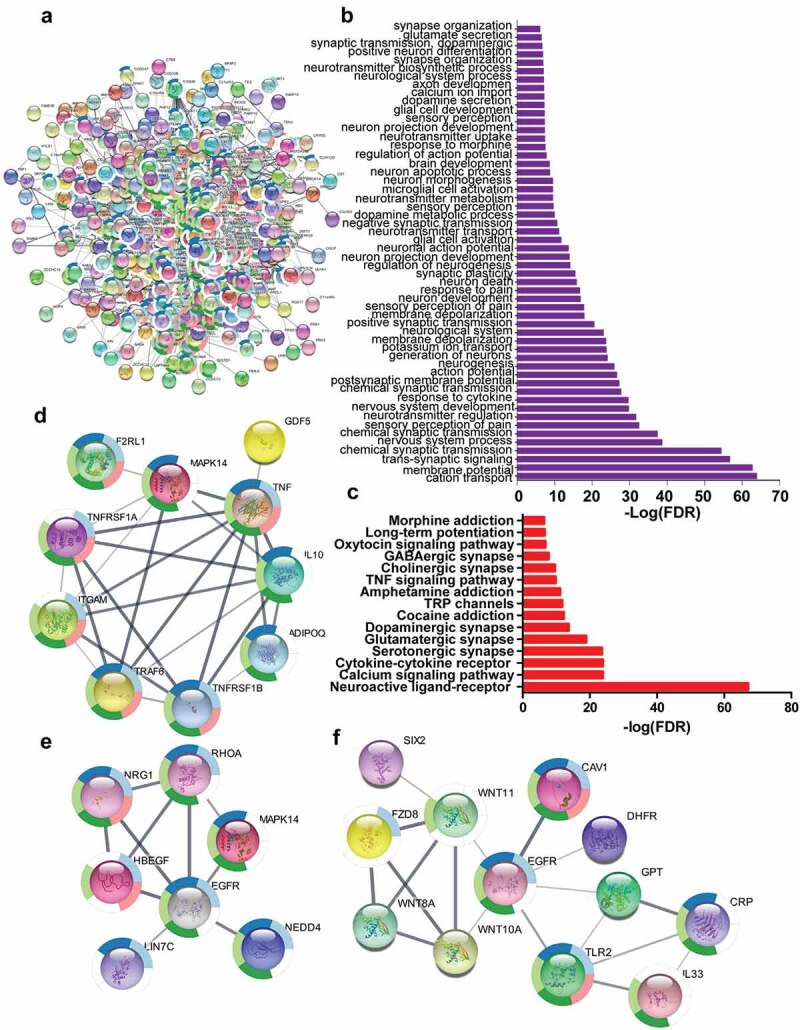
The paracrine factors from infiltrated dendritic cells targeted their receptors in neurons to sensitize pain. (a) The complicated network composed of genes associated with pain using String database to establish (b) and (c). GO process (b) and KEGG pathway (c) analysis indicated that genes were involved in the nerve activation associated with pain. (d–f) The complicated network contained the sub-networks associated with TNF-TNFRSF1A (d), NRG1-EGFR (e), and WNT10A-FZD8 (f) interaction, indicating that these paracrine factor–receptor interactions might participate in the regulation of pain.

Based on the String database, the network indicating interactions among proteins was established by using the genes which had been confirmed to participate in pain (Figure 6(a)). GO process (Figure 6(b)) and KEGG pathway (Figure 6(c)) analysis indicated that these genes were enriched in neuron active process including cation transport, membrane potential, neuroactive ligand–receptor interaction, and chemical synaptic transmission, etc., which were associated with pathological process of pain. The paracrine factors such as TNF, NRG1, and WNT derived from infiltrated dendritic cells, which had been identified to be likely involved in cancer pain, constituted sub-networks of the complicated network containing pain-related genes (Figure 6(d,e)). These sub-networks showed the interactions between paracrine factors and their receptors including TNF-TNFR (Figure 6(d)), NRG1-EGFR (ERBB) (Figure 6(e)), and WNT-FZD (Figure 6(e)) further indicated that paracrine factors TNF, NRG1, and WNT from infiltrated dendritic cells were involved in cancer pain.

### Activated signaling by paracrine factors regulated the expression of genes associated with pain

**Figure 7.**
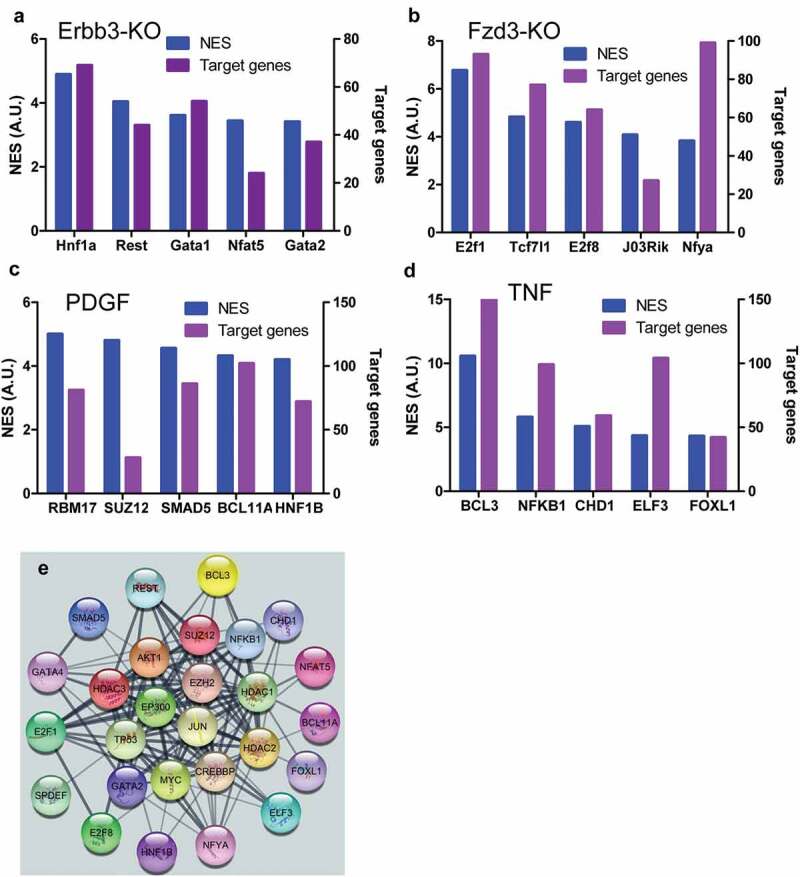
Top transcription factors activated by stimuli of paracrine factors. Top five transcription factors activated by NRG1-ERBB signaling and their target genes in NRG1-ERBB pathway (a), WNT-FZD signaling and the number of their target genes in WNF-FZD pathway (b), PDGF-PDGFR signaling and the number of their target genes in PDGF-PDGFR pathway (c), and TNF-TNFR signaling and the number of their target genes in TNF-TNFR (d), respectively. (e) The network composed of all top transcription factors. The network was established using String database. NES was the net expression score. A.U. was arbitrary unit. The numbers of target genes were indicated.

The top transcription factors activated by paracrine factor signaling were identified by using RNA-seq data from knockout, knockdown, over-expression of paracrine factors or their receptors, or paracrine factor-stimulated cells. Hnfa, Rest, Gata1, Nfat5, and Gata2 were identified as top five transcription factors activated by NRG1-ERBB signaling pathway using ERBB3 knockout RNA-seq data, and each transcription factor regulated dozens of genes including genes involved in pain (Figure 7(a)). E2f1, Tcf7l1, E2f8, 2310011J03Rik, Nfya were identified as top five transcription factors in the activated WNT-FZD signaling pathway by using FZD3 knockout RNA-seq data, and each transcription factor except for 2310011J03Rik regulated more than 40 genes among of genes which participated in pain (Figure 7(b)). RBM17, SUZ12, SMAD5, BCL, and HNF1B were identified as top five transcription factors activated by PDGF-PDGFR signaling pathway using RNA-seq data from PDGF-stimulated cells, and each transcription factor except for SUZ12 regulated more than 50 genes including some of the genes which were related to pain (Figure 7(c)). BCL3, NFKB, CHD1, ELF3, and FOXL1 were identified as top five transcription factors activated by the TNF-TNFR signaling pathway, and each transcription factor regulated more than 45 genes among of genes which were involved in pain (Figure 7(d)). These top transcription factors formed a complex network according to String database (Figure 7(e)), indicating that they would synergistically interact among these transcription factors when multiple signaling pathways were activated in the same cells such as nociceptor sensory neurons, which might synergistically regulate pain.

**Figure 8.**
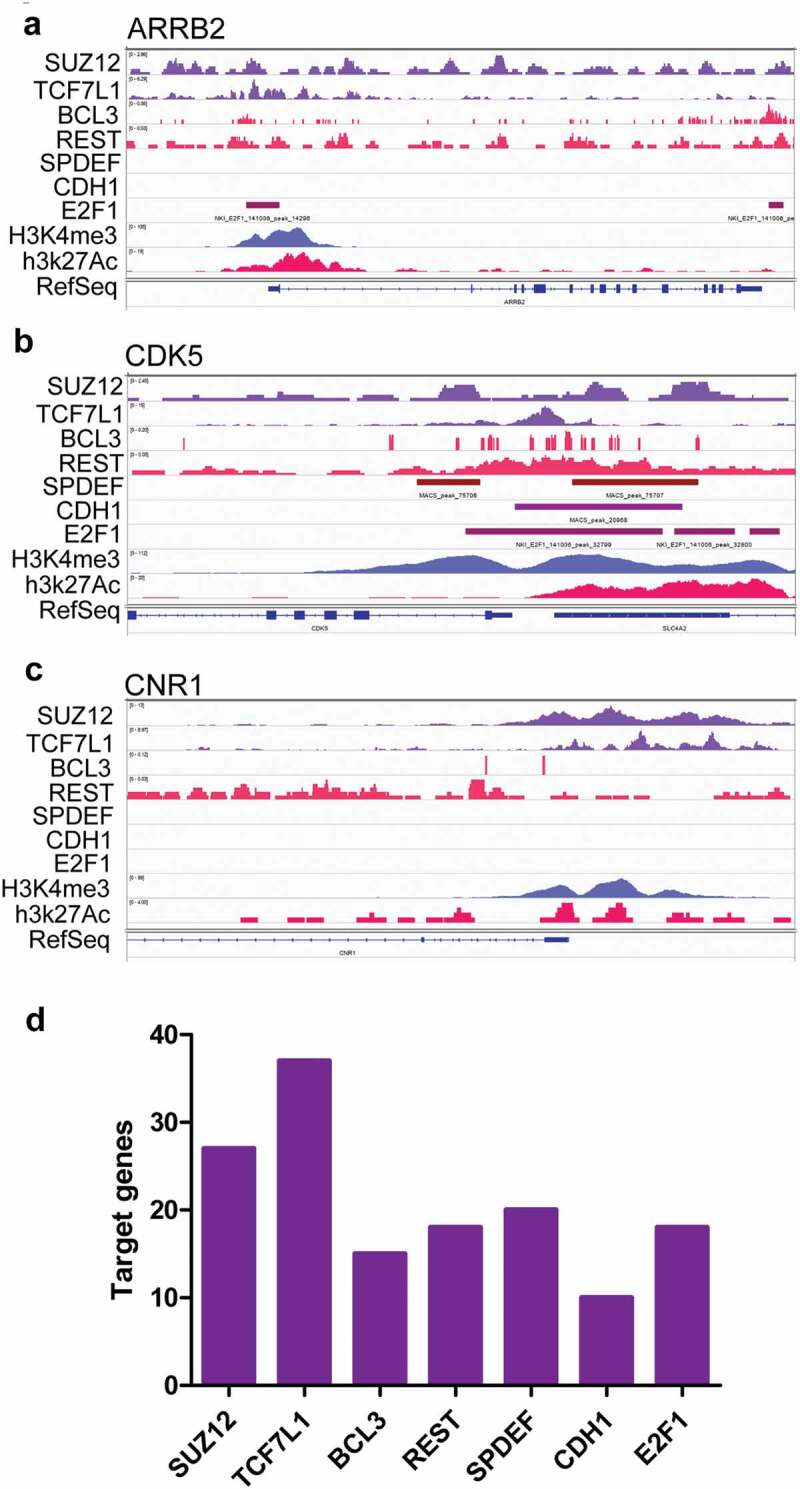
The top transcription factors activated by stimuli of paracrine factors regulated the expression of genes associated with pain. (a–c) ChIP-seq data indicated that the genes associated with pain such as ARRB2 (a), CDK5 (b), and CNR1 (c) could be regulated by some of the top transcription factors. (d) The numbers of genes associated with pain which could be regulated by the top five transcription factors.

Further ChIP-seq data were used to examine whether these transcription factors would really regulate the expression of genes associated with pain. H3K4Me3 and H3K27Ac peaks indicated the activated promoter region (Figure 8(a–c)). TCF7L1, BCL3, and E2F1 could bind to the ARRB2 promoter region, indicating that these transcription factors regulated the expression of ARRB2 (Figure 8(a)), which dampened the pain signaling mediated by stimuli of hormones or neurotransmitters. SUZ12, TCF7L1, BCL3, REST, SPDEF, CDH1, and E2F1 could bind to CDK5 promoter region to regulate the expression of CDK5 (Figure 8(b)), which was located at postmitotic central nervous system neurons, and functioned in diverse processes such as synaptic plasticity and neuronal migration. SUZ12, TCF7L1, and BCL3 could bind to the CNR1 promoter region to regulate the expression of CNR1 (Figure 8(c)), which was associated with anxiety and chronic pain. SUZ12, TCF7L1, BCL3, REST, SPDEF, CDH1, and E2F1 could regulate the expression of dozen of genes associated with pain (Figure 8(d)). These data indicated that paracrine factors derived from infiltrated dendritic cells could interact with their receptors in neurons or microglial cells to up-regulate the expression of genes associated with pain.

### Paracrine factors regulated neuropathic pain in multiple cancers

**Figure 9.**
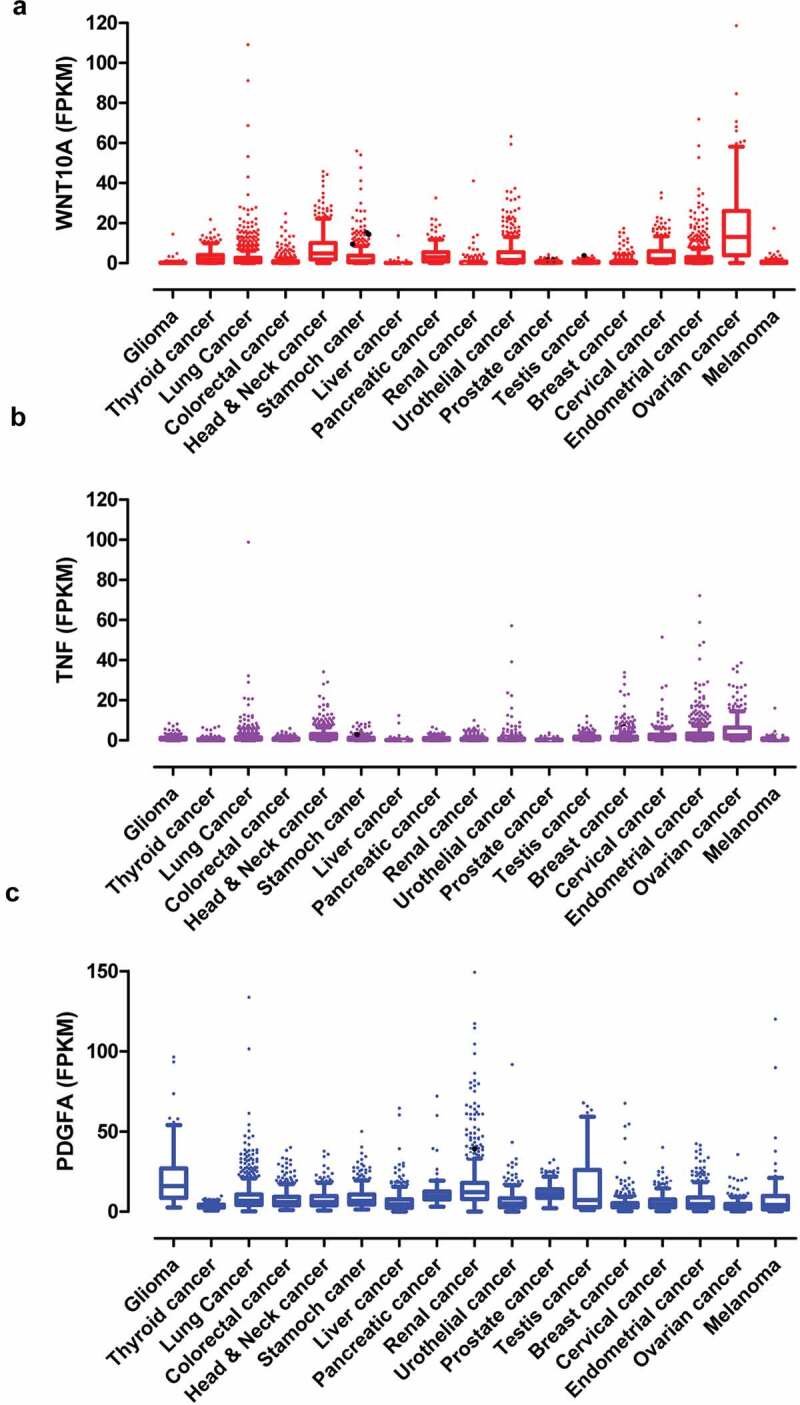
The paracrine factors were extensively expressed in various human tumors. (a–c) The expression of WNT10A (a), TNF (b), and PDGFA (c) in multiple human cancers. The data were from TCGA database. The box-and-whiskers plots indicated that some cancer patients had extremely high expression of WNT10A, TNF, and PDGFA that were shown as individual outlier symbols. D’Agostino and Pearson omnibus normality test indicated that the expression of paracrine factors did not distribute normally in all cancers.

WNT10A, PDGFA, TNF, and NRG1 had been identified as important paracrine factors from infiltrated dendritic cells to regulate neuropathic pain associated with lung adenocarcinoma. These factors were highly expressed in multiple cancers (Figure 9). According to RNA-seq data from various human tumors, WNT10A was highly expressed in lung cancer (2.9 ± 6.7 FPKM), head and neck cancer (7.3 ± 7.6 FPKM), stomach cancer (3.9 ± 7.3 FPKM), pancreatic cancer (4.3 ± 4.9 FPKM), urothelial cancer (4.9 ± 8.0 FPKM), endometrial cancer (3.4 ± 7.1 FPKM), and ovarian cancer (17.7 ± 17.9 FPKM), while the average FPKM of WNT10A in other cancers was less than 1.5 (Figure 9(a)). TNF was highly expressed in lung cancer (1.5 ± 3.9 FPKM), head and neck cancer (2.7 ± 3.7 FPKM), breast cancer (1.7 ± 2.7 FPKM), endometrial cancer (3.4 ± 6.7 FPKM), and ovarian cancer (4.9 ± 6.5 FPKM), while the FPKM of TNF in other cancers was less than 1.0 (Figure 9(b)). PDGFA was highly expressed in glioma (20.6 ± 16.6 FPKM), lung cancer (9.0 ± 9.0 FPKM), colorectal cancer (7.5 ± 5.5 FPKM), head and neck cancer (7.1 ± 5.4 FPKM), stomach cancer (8.9 ± 6.9 FPKM), liver cancer (6.3 ± 6.9 FPKM), pancreatic cancer (11.1 ± 7.6 FPKM), renal cancer (15.5 ± 14.3 FPKM), urothelial cancer (6.7 ± 7.0 FPKM), prostate cancer (11.8 ± 4.4 FPKM), testis cancer (16.0 ± 18.0 FPKM), breast cancer (16.3 ± 18.0 FPKM), and melanoma (8.9 ± 16.0 FPKM) (Figure 9(c)), while PDGFA in other cancers was less than 3.0 FPKM. Additionally, the box-and-whiskers plots indicated that WNT10A, PDGFA, and TNF were out of normal distribution due to extremely high expression in many tumor patients which were individually labeled with dots (Figure 9). D’Agostino and Pearson omnibus normality test indicated that the expression of WNT10A, TNF, and PDGFA in various cancers did not normally distribute. These data suggested that WNT10A, TNF, and PDGFA were involved in multiple types of cancer-mediated neuropathic pain, but probably not involved in each patient.

## Discussion

Neuropathic pain associated with cancers was caused due to tumor growth to squeeze or damage nerves, which would also be enhanced by inflammatory and growth factors through sensitizing nociceptor sensory neurons. Here the contributions of infiltrated dendritic cells educated by tumor microenvironment to neuropathic pain were unveiled by using RNA-seq, scRNA-sed, and ChIP-seq data, as well as bioinformatics assay. The results indicated that infiltrated dendritic cells generated pain-related hormones (direct regulation) or secreted paracrine factors (indirect regulation) to worsen neuropathic pain associated with multiple cancers.

### Dendritic cells located in tumor microenvironment synthesized and secreted pain hormones to regulate neuropathic pain

After dendritic cells infiltrated into lung adenocarcinoma and were educated via interaction with various cells including cancer cells in the tumor microenvironment, they were differentiated into CD1c^−^ and CD1c^+^ subpopulations [14]. The transcriptomes of both subtypes of dendritic cells had remarkable alterations including more than 1.5-fold increased expression of 2846 and 1313 genes compared with juxtatumor dendritic cells. The up-regulated genes were enriched in the pathways associated with pain such as histamine synthesis. Histamine promoted neuropathic pain via its receptors [24]. For instance, H1 and H2 knockout led to decreased responses to nociceptive stimuli [25,26]. This was consistent with the fact that histamine was released in response to tissue injury, and caused the sensitization of polymodal nociceptors resulting in increased firing rates which contributed to pain hypersensitivity [27]. This indicated that the dendritic cells educated by cell-cell communications or liquid factors in the tumor microenvironment could regulate neuropathic pain by synthesis and release of pain mediators such as histamine.

### *Paracrine factors sensitized* nociceptor sensory neurons to worsen neuropathic pain

The infiltrated dendritic cells educated by cell-cell communications or liquid factors up-regulated the expression of dozens of paracrine factors including cytokines, chemokines, and growth factors. Currently TNF, WNT10A, PDGFA, and NRG1 secreted by infiltrated dendritic cells in the tumor microenvironment were identified to be likely involved in the neuropathic pain using RNA-seq, scRNA-seq, and ChIP-seq data.

TNF was not only associated with neuropathic pain but also a potential target for the treatment of chronic pain [28]. Intra-sciatic injection of TNF in rats reproduced pain hypersensitivity that was similar to the neuropathic pain in humans [29,30]. Single-cell RNA-seq data indicated that TNFSF1was expressed in neurons, microglial cells, and endothelial cells in DRG, suggesting TNF might not only directly regulate neurons via its receptors but also indirectly regulate neurons by interacting with microglial cells or endothelial cells. Knockout of TNFR1 blocked that TNF enhanced the tetrodotoxin-resistant Na^+^ current in cultured DRG cells [31]. TNF signaling would activate transcription factors such as BCL3, CDH1, which might regulate the expression of genes associated with pain including CDK5. CDK5 was required in the nociceptive pathway [32]. Intrathecal administration of CDK5 inhibitor, roscovitine, attenuated formalin-induced nociceptive response [33]. Cdk5-knockout pups were unresponsive to noxious cutaneous pinch [34]. CDK5 mediated phosphorylation of TRPV1 at threonine-407 while conditional-knockout of primary nociceptor-specific Cdk5 reduced TRPV1 phosphorylation, resulting in significant hypoalgesia [35]. Thus, TNF derived from infiltrated dendritic cells in tumor microenvironment would likely promote neuropathic pain with cancers. The antibody against TNF or soluble TNFR might be used to relieve the pain associated with cancers [36].

Compared with TNF, the role of WNT10A in neuropathic pain was much less clear. WNT pathway was involved in neuropathic pain because the intrathecal injection of IWP-2, an inhibitor of WNT pathway, ameliorated neuropathic pain in CCI rats [37]. Actually, nerve injury and bone cancer could cause a rapid-onset and long-lasting expression of WNTs, which activated the WNT signaling pathway via their receptor FZDs in primary sensory neurons, spinal dorsal horn neurons, and astrocytes [38]. Moreover, spinal blockade of WNT signaling pathways could inhibit the production and persistence of neuropathic pain [38]. Consistent with the findings, scRNA-seq indicated that WNT receptors including FZD1 and 3 extensively were expressed on various neuron cells and non-neuron cells such as microglial and endothelial cells in DRG. RNA-seq data from FZD3 knockout mice indicated that the top transcription factors such as TCF7l1, E2F1 were activated by WNT signaling. ChIP-seq data indicated that TCF7l1 and E2F1 could regulate the expression of ARRB2. Loss of *Arrb2* resulted in prolongation of inflammatory and neuropathic pain and enhancement of GluN2B-mediated NMDA currents in spinal lamina IIo neurons [39]. These data suggested that WNT10A derived from infiltrated dendritic cells in tumor microenvironment aggravated neuropathic pain associated with cancers. The developed antibody against WNT receptors might be a candidate to block the WNT pathway to alleviate cancer pain [40].

PDGF signaling pathway was involved in neuropathic pain. PDGF α receptor (PDGFRα)/Fc chimera protein or PDGFR-dependent tyrosine kinase inhibitor AG17 suppressed thermal hyperalgesia and tactile allodynia induced by sciatic nerve ligation [41]. Injection of PDGF-BB into the paw produced nocifensive behavior in rats and led to thermal and mechanical pain hypersensitivity, which was associated with inhibition of M-current by PDFG signaling in DRG [1], although scRNA-seq data showed that PDFGR were mainly expressed in microglial cells. Activated microglial cells in the dorsal horn of the spinal cord were necessary for synaptic alterations in this region and pain hypersensitivity after nerve injury [42]. RNA-seq data from PDGF-stimulated cells indicated that transcription factors such as SUZ12, SMAD5, and BCL11 were activated by the PDGF signaling pathway. These transcription factors might regulate the expression of genes associated with pain such as TNF in microglial cells [43]. Thus, PDGF secreted by infiltrated dendritic cells in tumor microenvironment would regulate the neuropathic pain associated with cancer via activating microglial cells.

NRG1, a member of the epidermal growth factor of receptor tyrosine kinase family protein ligands (ERBB2, ERBB3, ERBB4), had about 30 isoforms generated by alternative splicing. Two major isoforms were membrane-bound and soluble isoforms of which the interaction with ERBB3 was essential for NRG1 functions [44]. Single-cell RNA-seq data showed that ERBB3 was mainly expressed in Sox10 positive neurons in DRG. RNA-seq data from ERBB3 knockout mice indicated that transcription factors including Hnf1a, Rest, and Gata1 were activated. ChIP-seq data indicated that Rest regulated CDK5 expression, which aggravated neuropathic pain as mentioned previously [32–35]. Additionally, Nrg1 regulated the pain after peripheral nerve injury by interacting with Errb2 on microglial cells [45]. Nrg1 derived from infiltrated dendritic cells in tumor microenvironment might be involved in neuropathic pain associated with cancers.

As mentioned above, cytokines such as TNF, growth factors including PDGFA and NRG1, and others like WNT10A were identified to interact with their receptors on neurons or other cells such as microglial cells to directly or indirectly sensitize neuropathic pain associated with cancers. In contrast, chemokines had been reported to aggravate pain associated cancers [46,47]. For instance, CXCL12-elicited bone pain induced by cancer while siRNA or inhibitor of CXCR4, a CXCL12 receptor, could antagonize CXCL12-elicited pain in spinal neurons [48]. Although CXCR4 was found to be highly expressed on DRG, the CXCL12 was not generated by infiltrated dendritic cells in the present research. CCL5 and CCL18 which were reported to participate in the neuropathic pain were massively synthesized by infiltrated dendritic cells although their receptors were not expressed on DRG [49,50]. Although chemokines derived from infiltrated dendritic cells were not confirmed to be involved in cancer pain in the current research, other cells such as macrophages in tumor microenvironment might generate massive CXCL12 to sensitize pain associated with cancers via interacting with CXCR4 in DRG.

### Paracrine factors-sensitized neuropathic pain extensively existed in various cancers

TNF, WNT10A, PDGFA, and NRG1 derived from infiltrated dendritic cells in lung adenocarcinoma microenvironment were proved to worsen neuropathic pain associated with cancers by sensitizing sensory neurons. Actually TCGA database indicated that these paracrine factors extensively existed in various cancers. For example, WNT10A was expressed in multiple tumors such as lung cancer, colorectal cancer, head and neck cancer, stomach cancer, pancreatic cancer, urothelial cancer, cervical cancer, and endometrial cancer. TNF was expressed on lung cancer, head and neck cancer, stomach cancer, urothelial cancer, breast cancer, cervical cancer, endometrial cancer, and ovarian cancer. PDGFA was expressed on glioma, lung cancer, colorectal cancer, head and neck cancer, stomach cancer, liver cancer, pancreatic cancer, renal cancer, urothelial cancer, cervical cancer, endometrial cancer, and melanoma. These data indicated the extensiveness that the paracrine factors secreted by infiltrated dendritic cells, which were educated by liquid factors or cell-cell commutation in the tumor microenvironment, promoted neuropathic pain associated with cancer via sensitizing sensory neurons. Thus, it might be a universe mechanism in neuropathic pain associated with multiple cancers. Inhibition of signaling induced by these paracrine factors might be a new strategy to alleviate cancer pain. Actually blockage of PDGF signaling could inhibit the pain mediated by acute inflammation [1].

However, it was notable that D’Agostino and Pearson omnibus normality test proved that the expression of WNT10A, TNF, and PDGFA did not distribute normally. As shown in box-and-whisker plots, some patients had extremely high expression while some patients had a very low expression. Perhaps the personalized diagnosis and intervention were essential for proper management of neuropathic pain in cancer patients, which would make each cancer patient receive the most effective and tolerable treatment for their individual pain management [51].

### Application and Perspective

A large amount of data from next-generation sequencing were deposited in different databases for freely using in academic research. However, it was a great challenge to dig up the meaningful bioinformation from these massive data. Here a successful sample was provided on how to integrate RNA-seq, scRNA-seq, and ChIP-seq data to unveil the novel factors and mechanisms to sensitize neuropathic pain associated with multiple cancers in the tumor microenvironment. The infiltrated dendritic cells in tumor microenvironment might not only synthesize hormones associated with neuropathic pain to directly control pain but also secret liquid factors such as TNF, PDGFA, WNT10A, and NRG1 to sensitize neurons so as to indirectly manage pain by up-regulated expression of pain-related genes via activating the transcription factors. These new findings supplied beneficial bioinformation to further design and develop new therapeutic medicines or strategies for cancer pain. Moreover, a similar strategy also could be used to investigate the contributions and new mechanisms of other cells such as macrophages in tumor microenvironment to neuropathic pain associated with multiple cancers.

## Conclusion

The current research identified that infiltrated dendritic cells in the tumor microenvironment could synthesize hormones and secret the paracrine factors such as TNF, WNT10A, PDGFA, and NRG1 which could sensitize sensory neurons to promote neuropathic pain associated with multiple cancers. The paracrine factors interacting with their receptors could cause the activation of downstream transcription factors such as BCL3, E2F1, SMAD5, and REST to up-regulated expression of CDH5, ARRB2, and other genes associated with pain. The blockage of paracrine factor signaling might be a new strategy to alleviate neuropathic pain associated with multiple cancers.

## Article highlights

- The infiltrated dendritic cells could synthesize hormones or secret paracrine factors.
- Interactions between paracrine factors and their receptors could up-regulate genes expression.
- The expression of a paracrine factors in various cancers did not distribute normally.
